# Duration of protection from live attenuated vs. sub unit HSV-2 vaccines in the guinea pig model of genital herpes: Reassessing efficacy using endpoints from clinical trials

**DOI:** 10.1371/journal.pone.0213401

**Published:** 2019-03-27

**Authors:** David I. Bernstein, Rhonda D. Cardin, Derek A. Pullum, Fernando J. Bravo, Konstantin G. Kousoulas, David A. Dixon

**Affiliations:** 1 Department of Pediatrics, University of Cincinnati College of Medicine, Cincinnati, OH, United States of America; 2 Division of Infectious Diseases, Cincinnati Children’s Hospital Medical Center, Cincinnati, OH, United States of America; 3 School of Veterinary Medicine, Louisiana State University, Baton Rouge, LA, United States of America; Rational Vaccines Inc, UNITED STATES

## Abstract

**Background:**

Although herpes simplex viruses (HSV) are a major target for vaccine development no vaccine is currently licensed.

**Methods:**

A live attenuated HSV virus vaccine, VC2 was compared to a subunit HSV vaccine, glycoprotein D (gD2) administered with the adjuvant, MPL/Alum using the guinea pig model of genital herpes. Three doses of intramuscular (IM) vaccine were provided followed by intravaginal challenge with HSV-2 at either 3 weeks or six months after the last vaccination.

**Results:**

Both VC2 and gD2 vaccines reduced acute genital disease. VC2 was somewhat more effective in reducing acute vaginal replication, the amount of virus in neural tissue, subsequent recurrent disease and recurrent virus shedding following challenge at 3 weeks post vaccination. Both vaccines continued to provide protection at 6 months after vaccination but the differences between the vaccines became more pronounced in favor of the live attenuated vaccine, VC2. Significant differences in acute disease, acute vaginal virus replication, recurrent disease and recurrent virus shedding (P<0.05 for each) was observed comparing the vaccines. Re-examination of protection for this study using criteria similar to those used in recent clinical trials (inclusion of recurrent disease) showed that efficacy may not be as high in this model as previously thought prompting a need to assess the best predictive outcomes for protection in humans.

**Conclusion:**

While both the live attenuated vaccine, VC2, and the gD2 subunit vaccine provided protection, the duration of protection appeared to be greater for VC2. Using the same evaluation criteria as used in human trials provided unique insights into the utility of the guinea pig model.

## Introduction

Genital herpes infections remain a major target for vaccine development [1, 2]. The most recent large trial of a herpes simplex virus type 2 (HSV-2) glycoprotein D (gD2) vaccine showed that it was effective against HSV-1 genital disease and infection but not HSV-2 genital disease or infection [3]. Thus, it appears that protection of the genital mucosa from HSV is possible and since HSV-1 genital infections are more common than genital HSV-2 in some areas [4, 5], this is a significant advance.

Animal models of genital HSV-2 infection including mice, rats and especially guinea pigs are commonly used to evaluate potential vaccines [2, 6]. These models have been criticized because protection is more easily achieved in animals than humans. However, the models differ from the clinical trials in several important ways. In most animal trials, high dose virus challenge is performed at the peak of immune responses, i.e. about 2–4 weeks after vaccination is completed [7–11] while in humans, subjects are followed for a year or more drastically increasing the time the vaccine must provide protection. Further, in the guinea pig model, the most common model used, protection from clinical disease is usually measured as prevention from death or lesion development during the acute period after challenge (about 2 weeks post challenge), while in clinical trials subjects are followed for years with the appearance of any lesions defined as a failure of the vaccine to protect.

Most vaccines used today can be broadly categorized as live or killed. In general, killed vaccines, including killed whole virus and subunit vaccines, are considered safer but live attenuated vaccines are thought to provide more long-lived durable protection. In this report, we compare two vaccines, a live attenuated HSV vaccine (VC2) [12–14] and a gD2 vaccine adjuvanted with MPL and alum [15, 16] which is similar to the vaccine used in the recent human trial [3] using the guinea pig model of genital herpes. One group of animals was intravaginally challenged with HSV-2 at 3 weeks and another group was challenged at 6 months after the last dose of vaccine to provide insight into the duration of protection. Protection from clinical disease was evaluated by comparing disease during the acute period (2 weeks post challenge), as is usually performed but we also evaluated protection over the entire period of observation (6 weeks post challenge), thus including animals that developed recurrent disease as is done in HSV vaccine clinical trials.

## Materials and methods

### Animals

Female Hartley guinea pigs (250–350 g, 4–6 weeks of age) were obtained from Charles River Breeding Laboratories (Wilmington, MA) and housed under AAALAC approved conditions at Cincinnati Children’s Hospital Medical Center. The protocol was reviewed and approved by the IACUC at Cincinnati Children's Hospital Medical Center.

### Vaccines

The gD2 vaccine was prepared by R. Eisenberg and G. Cohen (University of Pennsylvania) from Sf9 (*Spodoptera frugiperda*) cells infected with a recombinant baculovirus expressing gD2 (from HSV-2 strain 333) as previously described [16, 17]. Briefly, soluble gD2 (306) (truncated at the transmembrane domain (aa 1–306) was purified from baculovirus-infected insect cells (Sf9) as described previously [17]. For vaccine purification, the clarified and dialyzed medium was passed over a column of MAb DL6 coupled to Sepharose 4B, washed with 0.1 M Tris-0.15 M saline, pH 7.5 (TS), eluted with 0.1 M ethanolamine, concentrated by using a YM3 membrane (Amicon), and dialyzed against phosphate-buffered saline (PBS). The Alum/MPL adjuvant combination contained 50 μg of MPL (Sigma–Aldrich Corp, St. Louis, MO) and 200 μg of aluminum potassium sulfate (Sigma–Aldrich Corp, St. Louis, MO). To prepare the vaccine preparation, the gD2 was absorbed onto the Alum and then combined with MPL.

The VC2 recombinant virus was constructed utilizing the twostep double-Red recombination protocol implemented on the cloned HSV-1(F) genome [18] in a bacterial artificial chromosome (BAC) plasmid [19], as we have described previously [20, 21]. The VC2 virus contains two independent deletions; the gKD 31–68 deletion (37 amino acids (aa) in the amino terminus of gK t and a deletion of the amino-terminal 19 aa (4–22) of the UL20 gene. These two deletions are within domains that bind the amino and carboxyl terminal of gB rendering VC2 unable to enter via fusion of the viral envelope with cellular membranes[18, 22]. Next generation whole genome sequencing of VC2 revealed the presence of the gK (31-69aa) and UL20 (4-22aa) sequences in the UL53(gK) and UL20 genes. In addition, side-by-side sequencing and comparison to the HSV-1(F) parent sequence revealed the presence of 37 other nucleotide changes that did not result in amino acid changes [23].

### Experimental design

For evaluation of the clinical and virologic effects of prophylactic vaccination, 72 guinea pigs were randomized into three groups (N = 24/group): Group 1, Placebo: received no vaccine or adjuvant (received 10% sucrose); Group 2, received VC2; Group 3, received gD2 MPL/Alum. Animals were immunized IM in the upper thigh on days 63, 42 and 21 days prior to the 3 week viral challenge.

For gD2, animals were immunized with 500 μl containing 5 μg of gD2 while VC2 was administered at a dose of 1x10^6^ plaque forming units (pfu). Half of each group was challenged at 3 weeks after the last vaccine (standard challenge) and the other half, at 6 months after the last vaccination (late challenge).

One day before each viral challenge, animals were bled by toenail clip and the serum stored at -20°C for evaluation of neutralizing antibodies. Animals were inoculated with the challenge virus by rupturing the vaginal closure membrane with a moistened calcium alginate tipped swab (Calgiswab #3, Spectrum Labs, Los Angeles, CA) and instilling 0.1 ml of a virus suspension containing 1x10^6^ pfu of HSV-2 MS strain into the vaginal vault [15, 16]. Swab samples of cervicovaginal secretions were collected on days 2, 4 and 6 post inoculation (PI) and stored frozen (-80°C) until assayed for virus on Vero cells grown in BME (Gibco-Invitrogen) and 10% FBS (Hyclone, Thermo Fisher Scientific).

Guinea pigs were evaluated daily and primary genital skin disease quantified using a lesion score-scale ranging from 0 representing no disease to 4 representing severe vesiculoulcerative skin disease of the perineum [24]. Following recovery from primary infection, animals were examined daily from days 21–63 post each challenge for evidence of spontaneous recurrent herpetic lesions [24]. The number of lesion days (days on which a recurrent lesion was observed on the perineum) was recorded. Vaginal swabs were also obtained three days/week on days 21–63 post challenge to evaluate for recurrent virus shedding [15]. Swabs were stored frozen (−80°C) until they were processed for PCR analysis to determine the frequency of viral shedding into the genital tract. At the end of the follow-up period for each challenge, the guinea pigs were sacrificed, and the spinal cords and dorsal root ganglia (DRG) were harvested aseptically. These tissues were stored frozen (−80°C) until DNA was extracted from each animal for individual PCR evaluation of latent virus as previously described [25].

### Neutralizing antibody assay

To measure neutralizing antibody, the serum was heat-inactivated and a series of two-fold dilutions were prepared in titration medium as previously described [16]. HSV-2 MS strain (600 pfu) was added to each dilution, incubated for one-hour, and then plated onto Vero cells. After incubation for three days, the cells were stained. The final serum dilutions that produced a 50% reduction in the number of viral plaques compared to wells with no serum was used as the end-point. The end-point titer was calculated as the log_10_ of the dilution.

### qPCR of HSV-2 DNA

Viral DNA levels in DRGs and spinal cords harvested at the end of each study and vaginal swab samples collected between days 15–63 were determined [14, 26]. Briefly, DRG and spinal cords were homogenized on ice in 500 μl of 2% FBS BME. DNA was isolated from 200 μl of the tissue homogenates and vaginal swab media using QIAamp DNA Mini Kit (Qiagen #51306) according to the manufacturer’s protocol. The samples were incubated with Proteinase K for one hour at 65°C. Viral DNA was detected using primers specific for the HSV-2 gG gene yielding a 71 bp DNA product. The primer sequences (Sigma-Aldrich,St Louis, MO) were:

Forward: 5’-CGG/AGA/CAT/TCG/AGT/ACC/AGA/TC-3’; Reverse: 5’-GCC/CAC/CTC/TAC/CCA/CAA/CA-3’; and probe FAM-ACC/CAC/GTG/CAG/CTC/GCC/G-tamRA.

Each PCR reaction contained 50–100 ng of sample DNA, 50 μM of each primer, and 10 μL of Taqman Gene Expression Master Mix (ABI). A Tam/Fam fluorescent dye was used, and the PCR amplification was performed using a 7500 Fast Real-Time PCR system (ABI). Total volume of each sample was 20 μL. A standard curve was generated with 10-fold serial dilutions of purified HSV-2 DNA (ATCC) containing 10^5^ to 10^0^ HSV-2 copies in 50 ng of uninfected guinea pig DNA. The amplification program used included a pre-incubation step at 50°C for 2 min and at 95°C for 10 min, followed by 50 cycles consisting of a denaturation step at 95°C for 15 sec, annealing at 60°C for 1 min and elongation at 72°C for 10 sec. The limit of detection of the assay was between 10^0^ to 10^1^ copies. All samples which were negative for viral DNA were diluted ten-fold and the PCR assay was repeated.

### Statistics

For comparison of means, data were analyzed by ANOVA followed by a student’s t test comparison. The primary comparisons were placebo to each of the vaccines with a secondary analysis comparing each vaccine. Statistics were not adjusted for the multiple comparisons. Incidence data were compared by Fisher’s exact test. All comparisons are two-tailed. Data is presented as means and standard deviation.

## Results

We have previously shown that both gD2 [15, 16] and VC2 [12, 14] vaccines effectively limit acute virus replication, modify acute disease and decrease the number of subsequent recurrences. In this paper we extend our analysis to evaluate the duration of protection.

### Neutralizing antibody response

**Fig 1:**
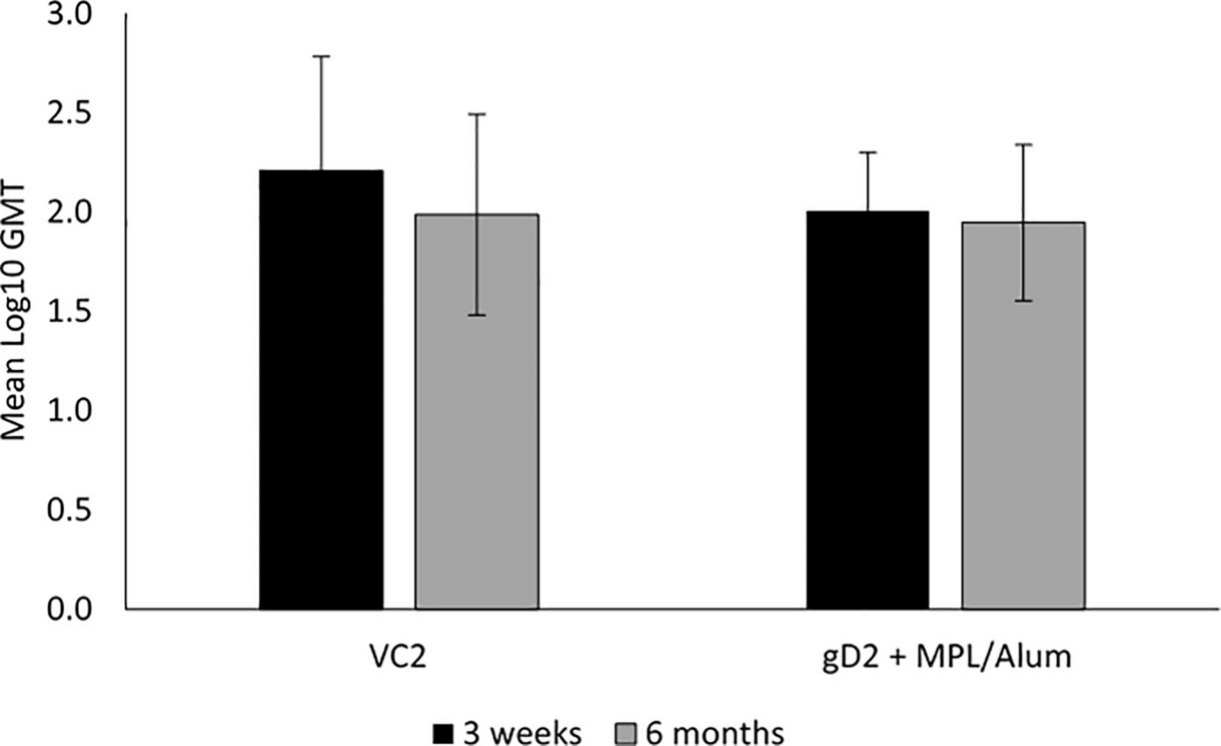
Neutralizing antibody titers induced by vaccination with the live virus vaccine, VC2 or gD2 MPL/Alum at either 3 weeks or 6 months after completion of a 3 dose series of IM vaccinations. Error bars are the standard deviation (SD).

At 3 weeks post the third vaccination, prior to the standard challenge, neutralizing antibody titers were detected in all vaccinated animals. A geometric meant titer (GMT) of 2.21 ± 0.58 in the VC2 vaccinated animals and 1.99 ± 0.51 for the gD2 group was detected. (Fig 1). Titers decreased slightly by 6 months, just prior to the delayed challenge to a GMT of 2.01 ± 0.29 for the VC2 groups and 1.95 ± 0.39 for the gD2 group.

### Standard virus challenge (3 weeks after the last dose of vaccine)

**Fig 2:**
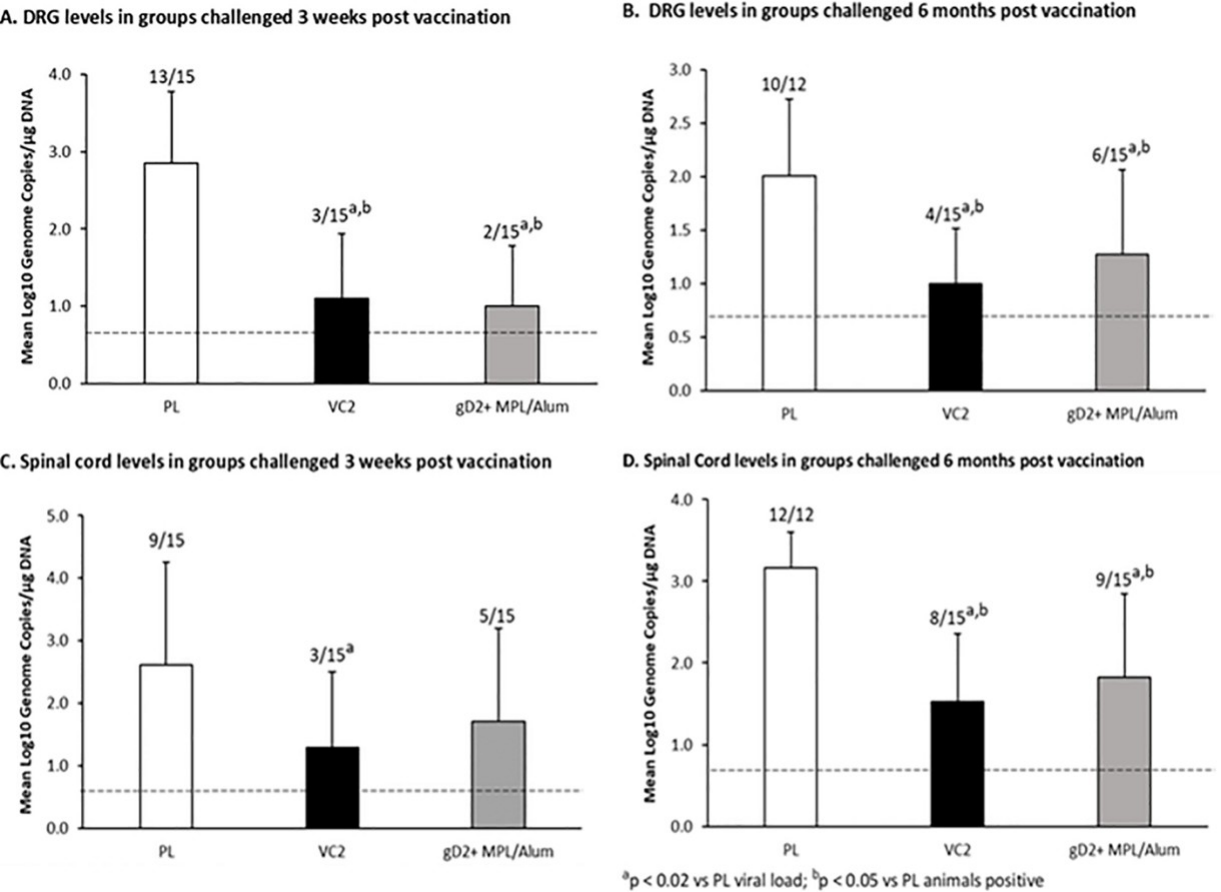
Effects of vaccination on HSV-2 DNA levels in the DRG and spinal cords at the completion of the observation period (day 63 post vaginal virus challenge). Panel A, levels in the DRG in the animals challenged at 3 weeks post vaccination, Panel B animals challenged at 6 months post vaccination, Panel C levels in the spinal cord in animals challenged at 3 weeks post vaccination, Panel D animals challenged at 6 months post vaccination. The number of animals with detectable virus DNA/total number animals evaluated are shown above each bar. Error bars are the standard deviation (SD).

Similar to our previous studies [14–16], both VC2 and gD2 reduced acute genital disease to a similar extent. Both significantly reduced the number of animals that developed any visual lesions and the severity of the acute disease (P<0.001 for each compared to placebo (Table 1). VC2 was somewhat more effective in reducing acute vaginal replication so that although significant (P<0.001) reductions were detected by both vaccines on days 2, and 6 compared to the placebo group (day 6 presented in Table 1). VC2 reductions were significantly greater than gD2 on days 2, 4 and 6 (P<0.03). Both vaccines also provided protection of the neural tissues reducing the number of animals with virus detected (P<0.05) and the quantities in the DRG (P<0.02) while only VC2 reduced the amount in the spinal cord (P<0.02). There were no significant difference between vaccines (Fig 2A–2C).

**Table 1:** Effect of vaccination on acute and recurrent genital HSV-2 infection in guinea pigs challenged intravaginally.

	Group	3 week challenge	P value	6 month challenge	P value
**Acute period**^**a**^					
% acute disease	PL	93		93	
	gD2	40	0.005 vs. PL	40	0.005 vs. PL
	VC2	33	0.002 VS. pl	07	< 0.001 vs. PL
			1.0		0.08 vs. gD2
Acute severity score					
	PL	10.0 ± 6.7		9.1 ± 4.4	
	gD2	1.0 ± 1.5	<0.001 vs. PL	1.6 ±2.1	<0.001 vs. PL
	VC2	0.8 ± 1.7	<0.001 vs. PL	0.3 ± 0.9	<0.001 vs. PL
			0.73 vs. gD		**0.04 vs. gD2**
Vaginal virus Replication (day 6)					
	PL	2.9 ± 0.5		3.3 ± 0.4	
	gD2	1.5 ± 0.7	<0.001 vs. Pl	2.0 ± 0.7	<0.001 vs PL
	VC2	1.0 ± 0.5	<0.001 vs. PL	1.0 ± 0.5	<0.001 vs PL
			**0.029 vs. gD**		**<0.001 vs gD2**
**Recurrent period**^**b**^					
% recurrent disease					
	PL	93		100	
	gD2	67	0.17 vs,. PL	93	1.0 vs. PL
	VC2	67	0.17 vs. PL	67	0.05 vs PL
			1.0 vs. gD2		0.17 vs. gD2
Recurrent days					
	PL	14.4 ± 12.5		9.0 ± 5.5	.
	gD2	3.2 ± 3.3	0.002 vs. PL	5.9 ± 3.7	0.09 vs PL
	VC2	1.1 ± 1.0	0.001 vs. PL	1.9 ± 1.9	<0.001 vs. PL
			**0.03 vs. gD**		**0.001 vs. gD2**

^a^ the acute period is the time form challenge to healing of the initial disease (days 1–14)

^b^ The recurrent period is from day 21 until the final observation on day 63.

Light grey highlights indicate significant differences vs. PL

Dark grey highlights indicate significant difference of VC2 vs. gD2

**Fig 3:**
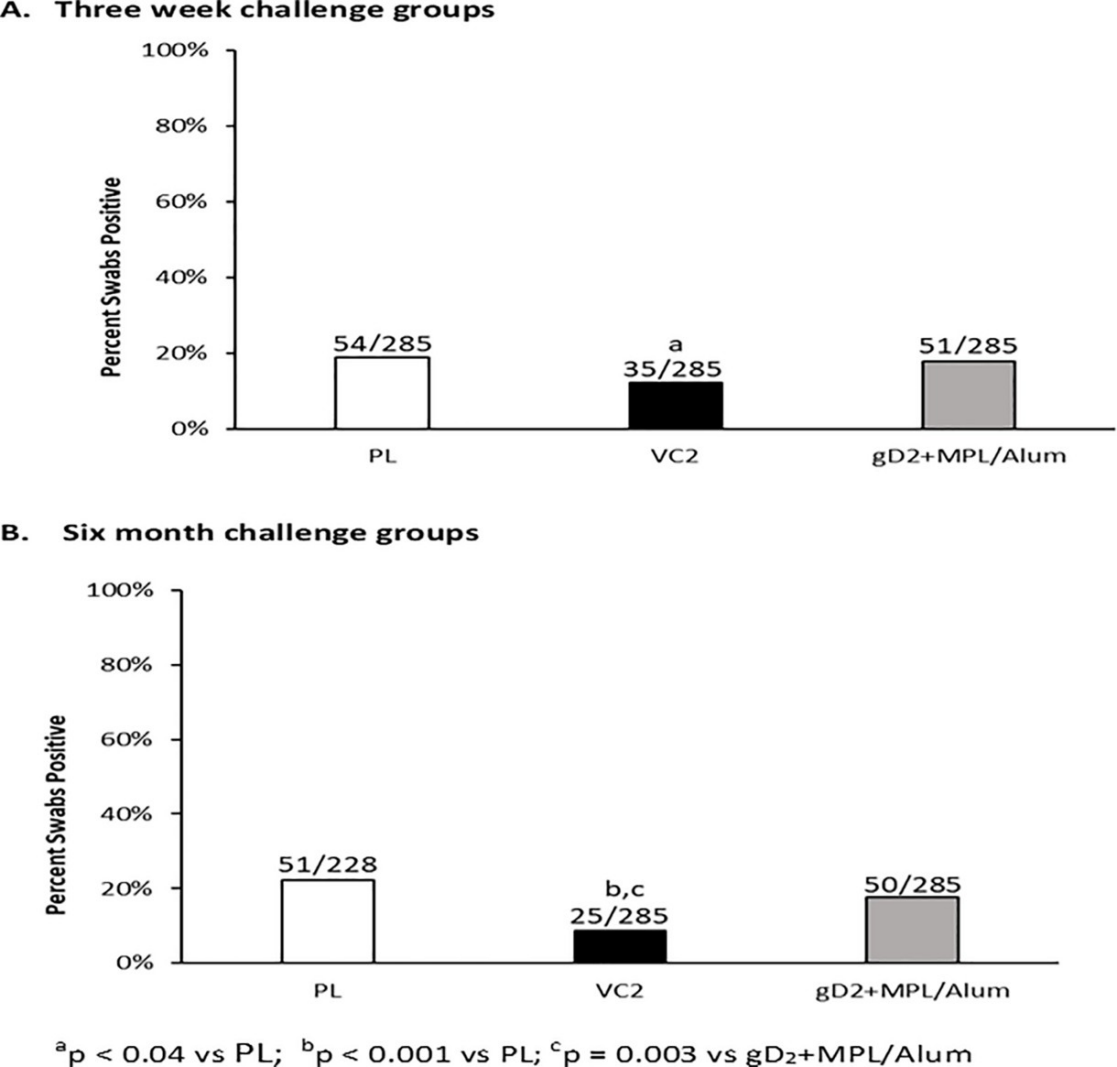
Efficacy of vaccination against recurrent vaginal virus shedding. After recovery from acute disease vaginal swabs were obtained three times per week to assess for HSV-2 shedding by qPCR. Panel A: shows the number of swabs that were positive/the total number of swabs obtained in the groups challenged three weeks post vaccination. Panel B shows the same data for the groups challenged six months after vaccination.

Subsequent recurrent disease was also reduced by both vaccines but VC2 further reduced the number of days with recurrent lesions compared to gD2 (P = 0.03, Table 1). Recurrent vaginal virus shedding has been the most difficult manifestation of HSV-2 to affect in the model. In this experiment, only VC2 reduced the number of days that HSV-2 was shed from the genital tract (P = 0.04, Fig 3A).

### Delayed virus challenge (6 months after the last dose of vaccine)

Both vaccines continued to provide protection at 6 months after vaccination but the differences between the vaccines became more pronounced in favor of the live attenuated vaccine, VC2. As seen in Table 1, both vaccines significantly reduced acute disease compared to the Placebo group with reductions in the number of animals with acute disease (P≤0.005) and the severity of the disease P<0.001), but the severity was lower for the VC2 compared to the gD2 group (P = 0.04). Similarly, vaginal virus titers were significantly reduced on days 2, 4 and 6 post challenge by each vaccine (P<,0.05 vs. Pl) but the reductions were significantly greater for the VC2 vaccine compared to the gD2 vaccine at each time point (P<0.03, day 6 shown in Table 1). Both vaccines also significantly reduced the number of animals with detectable viral DNA (P<0.05 vs Pl) and the levels in the DRG and spinal cord (P<0.02). These reductions were greater for VC2 although the differences were not significant comparing vaccines (Fig 2).

VC2 also provided a significantly greater reduction in recurrent disease (P = 0.05, Table 1) compared to gD2. Further, only VC2 reduced the days with recurrent virus shedding compared to Placebo with a significant difference between VC2 and gD2 (P = 0.003, Fig 3B). Similarly, only VC2 reduced the number of animals with recurrent disease (Table 1).

### Revaluation of protection

**Fig 4:**
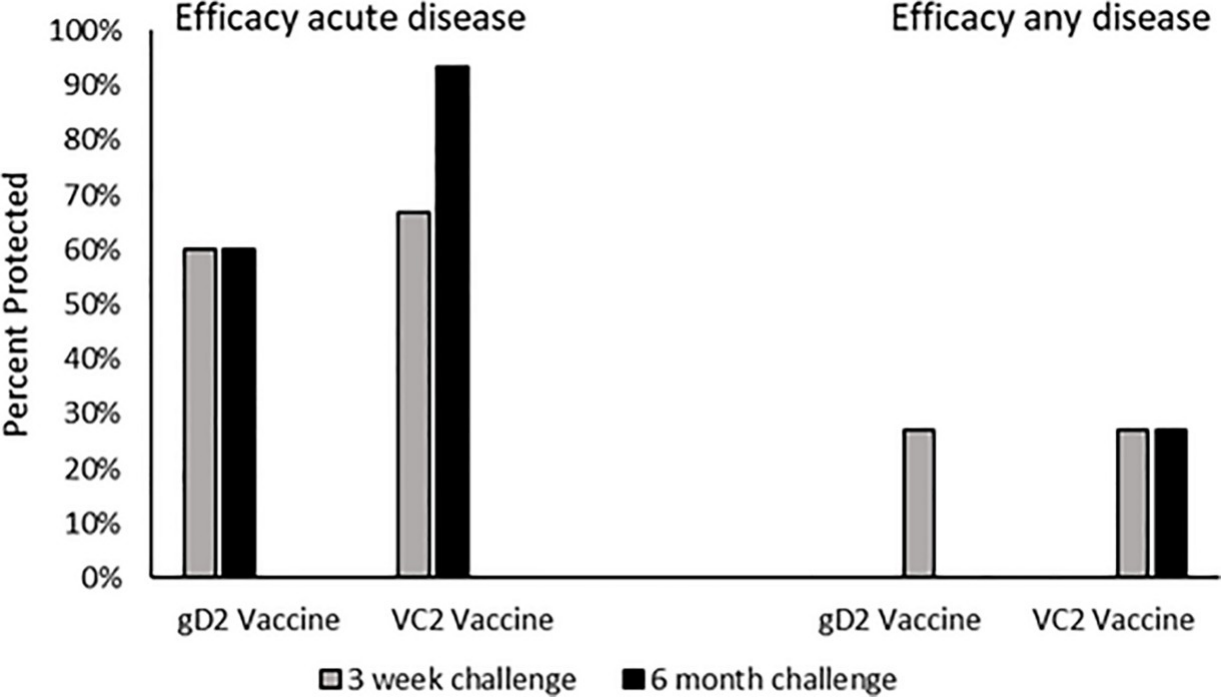
Efficacy of vaccination when evaluated against acute disease defined as development of genital lesions within two weeks of vaginal virus challenge or against any genital lesions that were detected during the observation period, seven weeks post vaginal virus challenge. The seven week observation includes recurrent lesions that develop after recovery from the acute disease.

To further assess the protection provided by the two vaccines, we evaluated efficacy using the definitions applied in the recent clinical trial [3], i.e. assessing lesion development over the entire period of observation. As seen in Fig 4, there are marked differences in the level of protection against genital disease during the acute period vs. the entire period of observation. While protection against genital lesions appears to be high during the acute period of observation, evaluation against lesion development over the entire period of observation is lower (<30% for either vaccine).

## Discussion

There is a general consensus that live vaccines offer more durable protection than killed or subunit vaccines. However, there are more concerns for the safety of live vaccines. Live attenuated vaccines for viruses that can become latent are perhaps of even more concern because if they persist there is always the possibility of reversion or recombination. Further, the recent link between HSV and neurodegenerative diseases, such as Alzheimer’s [27, 28], raises the possibility of a persistent vaccine virus acting as a risk factor. Therefore, VC2 was chosen as a live attenuated vaccine for these experiments as it was specifically designed to not enter nerve endings, reach the DRG or establish persistence [14, 29–31].

In the study presented here, the live attenuated vaccine, VC2, provided somewhat better protection than the subunit gD2 MPL/Alum vaccine when the HSV-2 challenge was done at the peak of the immune responses, 3 weeks after the third vaccination. Differences were observed in the reduction of acute vaginal replication, protection of the neural tissues and perhaps most importantly recurrent disease and recurrent virus shedding, two endpoints that are only available for study in the guinea pig model. When comparing protection provided at 6 month post vaccination, the differences between the two vaccines was even greater. VC2 provided more protection against acute disease, acute virus replication, virus levels in the neural tissue as well as recurrent disease and recurrent virus shedding compared to gD2. Of note, VC2 is based on the HSV-1(F) strain, and thus it is possible that a VC2 based vaccine expressing HSV-2 gD, or a similarly engineered live-attenuated HSV-2 VC2 vaccine may be even more protective. It should It should be noted that the gD2 protein used in the human trials was purified from the cell culture supernatant of CHO cells transfected with a truncated gD2 gene while the protein used in this and other[15, 16] guinea pig trials is derived from baculovirus-infected insect cells (Sf9), thus glycosylation will be different perhaps altering protective immune responses

In a previous experiment with delayed challenge at 6 months, there was no protection by gD2 MPL/Alum following 2 subcutaneous doses of vaccine (Bernstein and Cardin, unpublished). In that experiment, neutralizing antibody responses were not detectable at the 6 month challenge time point while in the experiment reported here, neutralizing titers decreased somewhat from their peak, but the GMT was still 2.01 ± 0.29 prior to the 6 month challenge. In the large clinical trial of gD2 Alum/MPL, the neutralizing titer decreased from a geometric mean concentration (95% CI) of 29.3 (26.3 to 32.7) to 6.9 (5.6 to 8.5) with a median value that was undetectable by month 16 [3] making it more similar to our previous experience with delayed challenge. Thus, despite the improved protection provided by gD2 MPL/Alum seen in the current experiment, VC2 still provided significantly better protection compared to gD2.

The guinea pig model of genital herpes mimics most aspects of acute and recurrent HSV disease in humans. Thus, it provides many endpoints that can be used to compare vaccines as we have shown in this manuscript. However, the model has been criticized because several vaccine including gD2 have provided protection in this model [7, 15, 32] but failed in clinical trials [3]. We show here, that if the same criteria for protection are applied in the model, the gD2 vaccine has a similar lack of protection. Thus, if the development of lesions at any point during the observation period, days 1–63, is used as criteria for success in the animals challenged 6 months after, vaccination the gD2 vaccine failed to provide protection. It should also be noted, that if the serologic criteria that was used in the human clinical trial, i.e. the development of antibody to HSV proteins other than gD are used in the guinea pig model, then over 50% of animals without acute disease have serologic evidence of HSV infection and thus would be assessed as vaccine failures [33]. However, when comparing protection, one should also consider that the challenge dose used in our model is almost assuredly more than occurs in human transmission, where shedding is rarely at the level of 10^6^ pfu [34, 35]. Thus, it is possible that complete vaccine protection can be overwhelmed in the models at these high challenge doses.

Of importance, using the criteria applied in human trials to the VC2 vaccine reveals that the vaccine was only minimally efficacious although it did provide better protection than gD2 MPL/Alum. It is unclear what criteria in the guinea pig model will best correlate to a successful human vaccine and thus definitive criteria awaits further human trials. We would suggest that at a minimum, vaccines should reduce acute disease, recurrences and perhaps infection of neural tissues. Thus, VC2 would meet these criteria and therefore we believe that VC2 warrants further investigation in human trials.

In summary, we have shown that the live attenuated vaccine VC2 provided more protection compared to a subunit gD2 MPL/Alum vaccine and that this difference became greater when the challenge was delayed until 6 months after vaccination, suggesting a longer duration of protection. Further, we provide evidence that when the same criteria that were used to evaluate HSV vaccines in the human trials are applied to the evaluation in guinea pigs, the gD2 vaccine also failed to provide protection. Identifying correlates of protection in animal models will provide improved guidance for the development of an effective vaccine for HSV disease. Similarly, if correlates are identified in human trials these should be evaluated in the guinea pig model and if reproduced, would further increase the utility of the model.

## Supporting information

S1 File NC3Rs ARRIVE guidelines checklist (fillable).pdf. (PDF)
